# Extramedullary plasmacytoma of the pancreas as an uncommon cause of obstructive jaundice: a case report

**DOI:** 10.4076/1752-1947-3-8785

**Published:** 2009-08-06

**Authors:** Pierre-Anthony Leake, Kathleen C Coard, Joseph M Plummer

**Affiliations:** 1 Department of Surgery, University Hospital of the West Indies, Mona, Kingston 7, Jamaica, West Indies; 2 Department of Pathology, University Hospital of the West Indies, Mona, Kingston 7, Jamaica, West Indies

## Abstract

**Introduction:**

Though uncommon, extramedullary plasmacytoma of the pancreas should be considered in the differential diagnosis of obstructive jaundice and pancreatic neoplasms. This report highlights a case of obstructive jaundice in a 46-year-old West Indian man that resulted from an extramedullary plasmacytoma.

**Case presentation:**

A 46-year-old West Indian man presented to our hospital with evidence of a significant upper gastrointestinal bleed. He gave a recent history of jaundice, constitutional symptoms and back pain. Ultrasonography revealed a mass in the head of the pancreas with resultant common bile duct dilatation. The patient required urgent surgical intervention for ongoing bleeding at which time a biopsy of the pancreas was taken. Histological analysis revealed a plasmacytoma of the pancreas. A blood film showing rouleaux formation and a skeletal survey demonstrating multiple lytic lesions confirmed multiple myeloma. Before further evaluation or treatment was carried out, the patient defaulted from follow-up and died from his illness seven months later.

**Conclusion:**

This case represents an example of multiple myeloma with visceral involvement, brought to clinical attention through involvement of the pancreas. The report serves to reaffirm knowledge of the various presentations, the optimal diagnostic tools and the current proposed treatment strategies for extramedullary plasmacytomas of the pancreas.

## Introduction

Extramedullary plasmacytoma represents an uncommon variant of plasma cell tumors involving organs outside the bone marrow. They are typically identified after the diagnosis of multiple myeloma. The following case report describes such a tumor involving the pancreas, presenting with obstructive jaundice and mimicking an adenocarcinoma of the pancreas.

## Case presentation

A 46-year-old West Indian man presented to our emergency department with a two-day history of intermittent passage of blood from the rectum. This was associated with coffee-ground vomitus and symptoms of anemia. He gave a past history of peptic ulcer disease. He had no history of non-steroidal anti-inflammatory drug (NSAID) use. Three weeks previously, the patient had noted jaundice associated with pruritus, dark urine, constitutional symptoms and back pain. Examination revealed the patient to be anemic, jaundiced and hemodynamically normal. Minimal epigastric tenderness was elicited and a Courvoisier's gall bladder palpated. Digital rectal examination revealed altered blood, and nasogastric drainage revealed coffee-ground material.

A diagnosis of an upper gastrointestinal bleed secondary to peptic ulcer disease was made. However, diagnoses of periampullary pancreatic carcinoma or locally advanced gastric carcinoma were also considered. Initial blood investigations revealed a hemoglobin level of 8.9 g/dL. Coagulation studies and electrolytes were normal. Liver function tests suggested obstructive jaundice. Abdominal sonography showed a normal liver with a distended (109 mL) gall bladder and dilated biliary system. The pancreas appeared bulky and heterogeneous.

Further evaluation of the pancreatic lesion was planned. However, our patient's condition became complicated by persistent bleeding from the rectum and a resultant fall in his hemoglobin level to 4.4 g/dL over a 2-day period. Though he remained hemodynamically normal, limited blood availability, continued bleeding and the unavailability of endoscopy prompted a decision to proceed to laparotomy. At laparotomy, an actively bleeding ulcer was identified in the first part of the duodenum and it was oversewn with good effect. A bosselated mass, not related to the ulcer, was noted occupying the head and neck of the pancreas. There was no evidence of local invasion. A biopsy of the pancreatic mass was taken.

**Figure 1:**
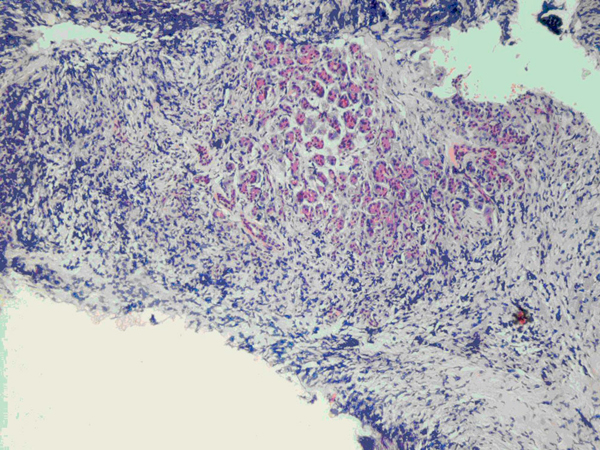
**Low-power view of pancreatic biopsy showing residual pancreatic acini centrally, surrounded by a dense infiltrate of smaller cells (hematoxylin and eosin stain, ×100)**.

**Figure 2:**
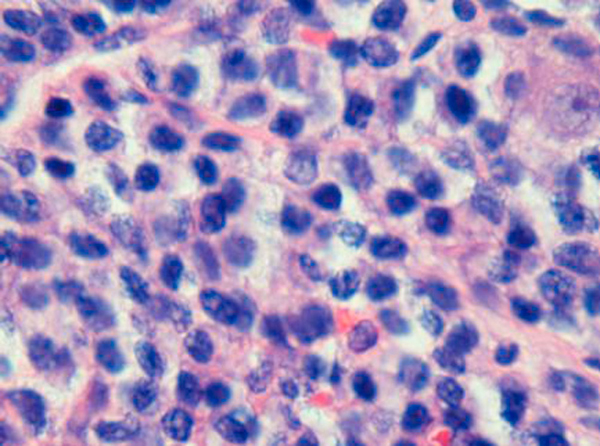
**High-power view of the pancreatic biopsy exhibiting characteristic nuclear features of plasma cells (hematoxylin and eosin stain, ×400)**.

**Figure 3:**
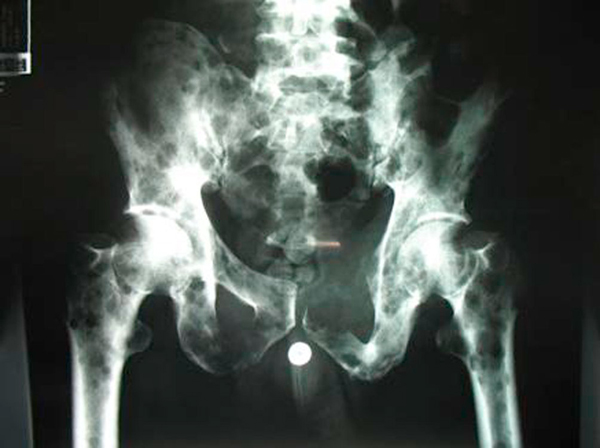
**Plain radiograph of the pelvis and proximal femora showing extensive lytic lesions**.

The patient's postoperative course was uneventful and he was discharged five days postoperatively. Histological analysis of the pancreatic biopsy revealed plasmacytoma (Figures 1 and 2). Following this diagnosis, appropriate investigations for multiple myeloma were initiated in the outpatient clinic. Rouleaux formation was noted on blood film, and lytic lesions were noted in the skull, pelvis and proximal femora (Figure 3), both humeri, scapulae, clavicles and in multiple ribs. A complete collapse of the 7th thoracic vertebra was also evident. Based on these findings, the patient was re-evaluated as having multiple myeloma with extramedullary plasmacytoma of the pancreas. Unfortunately, the patient defaulted from follow-up before further investigation and treatment, including bone marrow biopsy, and died from his illness seven months after initial presentation.

## Discussion

Multiple myeloma is a malignant hematologic neoplasm characterized by the uncontrolled proliferation of clonal plasma cells and accounts for 10% of malignant hematologic neoplasms and 1% of neoplasms overall [1]. Typically, multiple myeloma is a disease of middle-aged and older people with peak incidence in the 6th and 7th decades of life. Its predominant bone marrow involvement results in anemia, hypercalcemia with renal impairment and impaired immunity. This skeletal involvement results, classically, in 'punched out' lytic lesions on radiography [2].

A plasmacytoma represents a discrete, often solitary, mass of neoplastic plasma cells which may occupy medullary or soft tissue, that is, extramedullary, sites [3]. Extramedullary plasmacytomas account for 5% of all plasma cell neoplasms [4]. They are more commonly related to underlying multiple myeloma. Rarely, there may be no evidence of bone marrow involvement (<5% plasma cells and normal bone scan), with the plasmacytomas occurring either *de novo* or secondary to another extramedullary plasmacytoma.

The most frequent sites of extramedullary plasmacytomas are the nasal fossae and other parts of the upper respiratory tract. Other typical extraosseous sites of plasmacytoma include the liver, spleen and lymph nodes. When the pancreas is involved, it may be the sole site [5,6] or there may be coexistent involvement of other organs such as the stomach and bladder [2]. To date, just over 20 clinical cases of extramedullary plasmacytomas involving the pancreas have been reported in the English language literature. Most have been identified in patients already known to have underlying multiple myeloma or other extramedullary plasmacytomas. In our patient, the diagnosis of multiple myeloma was made immediately following the diagnosis of the plasmacytoma of the pancreas.

The typical presentation of extramedullary plasmacytomas of the pancreas includes jaundice and abdominal pain, often related to obstruction of the biliary tree. The radiologic features are non-specific. Ultrasonography typically demonstrates a heterogeneous focal mass which is hypoechoic relative to the normal parenchyma. It is most often located in the head of the pancreas [7]. Computed tomography (CT) findings of a focal multilobulated mass with homogeneous intravenous contrast enhancement have been described more commonly, though diffuse pancreatic enlargement may be appreciated [6]. Dual phase acquisition CT with pancreatic protocol is considered the investigation of choice as it demonstrates improved detection of hypovascular and hypervascular lesions [2]. Magnetic resonance imaging (MRI) has limited documented use for the detection of extramedullary plasmacytomas of the pancreas, though some authors suggest that this modality may be better in demonstrating pancreatic infiltration than CT [6]. It is felt that the development of fast CT scans with multiphasic capabilities, particularly in the arterial phase, will improve the detection of extraosseous manifestations of multiple myeloma [2].

The use of endoscopic retrograde cholangiopancreatography (ERCP) in the diagnosis has not been reported widely. Abu-Hammour *et al.*[8] noted biliary obstruction related to a mass in the head of the pancreas. ERCP demonstrated smooth strictures, dilated common bile duct and proximal pancreatic duct. Hirata *et al.*[4] suggested that the demonstration of a smooth stenosis of the biliary tree was more suggestive of a plasmacytoma than adenocarcinoma where irregular stenosis is classical. The definitive diagnosis is usually confirmed by open biopsy as the risk of dissemination of malignant cells with percutaneous biopsy has made this route unpopular [4].

Conventional treatment for multiple myeloma involves steroid-chemotherapy combinations and radiotherapy for symptomatic lesions. Newer modalities being investigated include thalidomide, antiangiogenic agents and stem cell transplantation [9]. There appears to be no standardized treatment for extramedullary plasmacytomas of the pancreas however. External beam radiotherapy, chemotherapy and bypass procedures, alone or in combination, have been described. Owing to the highly radiosensitive nature of plasma cell tumors, radiation therapy has been suggested to be the treatment of choice [4]. Surgical procedures, other than distal pancreatectomy for isolated pancreatic tail involvement, are not commonly performed owing to the often systemic nature of the disease and the radical nature of these surgical procedures [4]. Unsuspected cases may undergo pancreatic resection typical for pancreatic lesions with subsequent identification of plasma cells [10]. Chemotherapeutic agents are commonly used, particularly when plasmacytomas are secondary in nature. Of the cases mentioned in the literature, only one patient had therapeutic surgical resection, four had chemotherapy alone, five had radiotherapy alone and three had combination radiotherapy and chemotherapy [4,11]. Of those patients who underwent radiotherapy or chemotherapy, resolution of biliary obstruction was noted in the majority thus avoiding the need for surgical bypass or stenting [4,11]. Survival in these patients is related to the underlying systemic illness. Consequently, the course for extramedullary plasmacytoma not related to multiple myeloma is more favorable than that of multiple myeloma or solitary plasmacytoma of the bone. Life expectancy related to extramedullary plasmacytoma of the pancreas has been quoted as 1 day to 6 years in the literature [4].

In our patient, even if resolution of obstructive jaundice was achieved with the institution of radiotherapy and chemotherapy, his overall prognosis would have been guarded based on the systemic component of the disease.

## Conclusion

This report represents a rare case of extramedullary plasmacytoma of the pancreas. It re-emphasizes the need to consider all possible differentials when evaluating pancreatic masses. Owing to its uncommon nature, the comparative assessment of treatment modalities is not feasible. In the absence of such evidence, extrapolation from the treatment of multiple myeloma seems reasonable and has been shown to improve the extent of obstructive jaundice in these patients.

## Abbreviations

CT: computed tomography; ERCP: endoscopic retrograde cholangiopancreatography; MRI: magnetic resonance imaging; NSAID: non-steroidal anti-inflammatory drug.

## Consent

Informed consent was obtained from the patient's next-of-kin for publication of this case report and the accompanying images. A copy of the written consent is available for review by the Editor-in-Chief of this journal.

## Competing interests

The authors declare that they have no competing interests.

## Authors' contributions

PL was integral in the management of the patient, carried out the surgical procedure and literature review and primarily wrote the manuscript. KC performed the pathological examination and assisted in the revision of the manuscript. JP was a major contributor in the writing of the manuscript and provided guidance through the process. All authors read and approved the final manuscript.

## References

[B1] Kyle RA Rajkumar SV Multiple myeloma Blood 2008 111 2962 2972 10.1182/blood-2007-10-078022 18332230 PMC2265446

[B2] Kazama T Ng CS Giralt SA Multiphasic CT and MRI appearances of extramedullary multiple myeloma involving the stomach, pancreas, and bladder Clin Imaging 2005 29 263 265 10.1016/j.clinimag.2004.11.002 15967318

[B3] Nolan KD Mone MC Nelson EW Plasma cell neoplasms: Review of disease progression and report of a new variant Surg Oncol 2005 14 85 90 10.1016/j.suronc.2005.05.001 15993050

[B4] Hirata S Yamaguchi K Bandai S Izumo A Chijiiwa K Tanaka M Secondary extramedullary plasmacytoma involving the pancreas J Hepatobiliary Pancreat Surg 2002 9 111 115 10.1007/s005340200012 12021905

[B5] Fischer A Suhrland MJ Vogl SE Myeloma of the head of the pancreas: a case report Cancer 1991 67 681 683 10.1002/1097-0142(19910201)67:3<681::AID-CNCR2820670325>3.0.CO;2-J 1985760

[B6] Balliu E Casas JD Barluenga E Guasch I Multifocal involvement of the pancreas in multiple myeloma: sonographic, CT, and MR imaging findings AJR Am J Roentgenol 2003 180 545 546 12540476 10.2214/ajr.180.2.1800545

[B7] Mitchell DG Hill MC Obstructive jaundice due to multiple myeloma of the pancreatic head: CT evaluation J Comput Assist Tomogr 1985 9 1118 1119 10.1097/00004728-198511000-00024 3902920

[B8] Abu-Hammour AM Venu RP Etzkorn KP Showel JL Zaytsev PM Brown RD Common bile duct obstruction caused by multiple myeloma of the pancreas Gastrointest Endosc 1996 44 606 608 10.1016/S0016-5107(96)70018-4 8934171

[B9] Smith A Wisloff F Samson D UK Myeloma Forum Nordic Myeloma Study Group British Committee for Standards in Haematology Guidelines on the diagnosis and management of multiple myeloma Br J Haematol 2006 132 410 451 10.1111/j.1365-2141.2005.05867.x 16412016

[B10] Deguchi Y Nonaka A Takeuchi E Funaki N Kono Y Mizuta K Primary pancreatic plasmacytoma Am J Clin Oncol 2004 27 247 249 10.1097/01.coc.0000092613.05046.28 15170142

[B11] Hiller N Goitein O Ashkenazi YJ Plasmacytoma of the pancreas Isr Med Assoc J 2004 6 704 705 15562814

